# Predictors of Healthcare Professionals’ Work Difficulty Perception during the COVID-19 Pandemic: Study of Work Environment in a Pandemic Hospital

**DOI:** 10.3390/ijerph19095174

**Published:** 2022-04-24

**Authors:** Abdurrahim Emhan, Safa Elkefi, Onur Asan

**Affiliations:** 1 Collage of Business Administration, University of Central Florida, Orlando, FL 32816, USA; abdurrahim.emhan@ucf.edu; 2 Stevens Institute of Technology, School of Systems and Enterprises, Hoboken, NJ 07030, USA; selkefi@stevens.edu

**Keywords:** performance, work environment, job difficulty, emotional wellbeing, depression, burnout, COVID-19, pandemic hospital, health care professionals, doctors, nurses, information availability, motivation, social support

## Abstract

COVID-19 has dramatically changed the work environment in healthcare, which is creating an additional burden for healthcare professionals. In this study, we investigate the factors that trigger professionals to have negative perceptions of their jobs during the pandemic. A cross-sectional survey is used for this study. The respondents are selected based on convenience random sampling. We use 345 questionaries for the analysis. Respondents are health care professionals (nurses, doctors, midwives, technicians, etc.) working in a pandemic hospital in Turkey. We run a multivariable logistic regression model to analyze the predictors of work difficulty perception. The model is adjusted for the respondents’ demographical characteristics and emotional wellbeing. We found that depression and burnout are significantly correlated with the perception of job difficulty (OR Severe PHQ-9 = 10.8, *p* = 0.004; OR Severe Burnout = 7.83, *p* < 0.001). The professionals who are changed from one department to another are also more likely to perceive the job as difficult (OR Department Change = 1.60, *p* = 0.045). However, the professionals that received sufficient applause from society are more likely to think that they did not face any difficulties doing their job during the pandemic (OR Applause = 0.56, *p* < 0.016). Anxiety, monetary motivation, religious beliefs, and information availability did not contribute to the perceived difficulty in their jobs. Thus, efforts need to be made to give them more social support and smooth their changes in departments and functions to facilitate their jobs.

## 1. Introduction

Human resources represent the largest cost in the global healthcare system. Work motivation and the relationship between job motivation and individual performance are one of the key issues investigated in studies on organizational behavior and human resources (HR) management [1]. In contrast to industrial management, healthcare management is vastly different [2]. Among medical employees, work motivation is significantly important. Doctors treat patients who require special care and attention. The practitioner must be committed and dedicated as well as be mentally strong to cope with difficult situations with patients [3]. The World Health Organization suggests that the main determinant of healthcare quality is the motivation of healthcare professionals [4].

Many factors can impact health care providers’ motivation. One study reports that the most important motivation factors for medical professionals are work achievements, followed by remuneration, cooperation, and work characteristics [5]. Another study indicates that the work environment and independence level at work were highly correlated with the job satisfaction of doctors and nurses [6]. In addition, in healthcare, a positive work environment is critical to the delivery of high-quality patient care [7]. A positive attitude towards work has been strongly linked to attracting and retaining healthcare professionals [8]. However, there has been little attention paid to health-related tasks and the contextual factors in the work environment that impact the performance of workers [9].

Crises situations put work environments under pressure and pose multiple challenges to healthcare workers (HCWs). In epidemics and pandemics, healthcare professionals are at an increased risk regarding their physical and mental health [10]. Many factors may contribute to the deterioration of workers’ mental health, such as excessive workload due to a rise in infection rates, defective personal protective equipment, and insufficient access to hospital beds [11]. Other causing factors are lack of preparation and preparedness, and emotional distress risen from fear of illness [12]. Globally, new data suggest that HCWs are woefully unprepared to respond to a pandemic [13]. In addition, HCWs’ pressure can result from the goals they are trying to achieve, such as reducing the spread of infection, developing appropriate short- and long-term strategies, and formulating long-term plans [14].

We have been facing a significant health crisis as a result of the new Coronavirus 19 (COVID-19) since March 2020. The World Health Organization (WHO) estimates that over 10% of global infectious diseases are attributable to healthcare workers (HCWs) [15]. Compared to previous epidemics, this virus has been more challenging because of the lack of established knowledge and treatments and the high contagiousness [16]. Globally, healthcare workers and systems have been put under unprecedented strain by the COVID-19 pandemic. Pressures of this kind may adversely affect working conditions, psychological well-being, and perceptions of safety [17]. More studies are needed to better understand the relationship between job difficulty and COVID-19 mediating changes in work conditions. In this study, we investigate the impact of work environment-related factors on the perception of job difficulty for HCWs actively working during the COVID-19 crisis in a pandemic hospital.

## 2. Materials and Methods

### 2.1. Study Design

The study was approved by the internal review board of Gazi Yaşargil Education and Research Hospital’s Ethics Committee (IRB ID 677). We designed a survey with 75-item questions (excluding demographic questions). The data collection occurred between March 2020 and April 2020. We distributed a paper-based survey in the hospital to 700 healthcare workers including doctors, nurses, midwives, health technicians, and other staff and received 344 responses. Our data collection happened in a pandemic public hospital in Diyarbakir province, Turkey, secluded for infected patients. This hospital had 87,355 positive COVID cases between March 2020 and March 2021, and approximately 15,000 reported COVID cases during the time of the study. The hospital also had hundreds of their workers infected and three of the providers died during the study period.

### 2.2. Instrumentation

The study adopted a well-established and validated survey questions from various scales. All survey questions were modified and adapted to fit the context of our research. For this study, the survey was designed to assess healthcare workers’ work conditions, work stress, interpersonal conflict, and difficulty of work perception. It also recorded respondents’ demographic information such as gender, age, education level, the position of the worker, and their department. The age was categorized into two subgroups with a mean cutoff (m = 34.6). HCWs who are less than ≤34 years old and HCWs who are more than ≥35 years old [18]. The Depression scale, the Anxiety scales, the Burnout scale and the Turnover scale were, respectively, developed by Kroenke et al. [19], Spielberger et al. [20], Kristensen et al. [21] and Walsh et al. [22]. The responses to the S-Anxiety (STAI-I) scale assess intensity of current feelings “at the moment of the answer” and the T-Anxiety (STAI-II) scale assess the frequency of the feelings “in general”.

STAI-I and STAI-II have 20 questions each with 4 Likert scale. The resulting scores ranged both from 20 to a maximum of 80. STAI scores are commonly classified as “no or low anxiety” (20–37), “moderate anxiety” (38–44), and “high anxiety” (45–80) [23]. The Copenhagen Burnout Inventory scale is a 7-question scale. It is often interpreted in a 3-group classification (<14: low, 14–20: medium and >20: high burnout) [24]. The PHQ-9 is a 9-depression question scale. A severity classification is commonly used to interpret the PHQ-9 scale as 3 groups (5–9: low, 10–14: moderate, and 15–27: high) [25].

The job difficulty was assessed using the following question: “How much difficulty did you face doing your job, handling tasks, or interacting with other people?” A forced 4 Likert scale was used to assess the job difficulty perception with 0 “Not at all difficult” and 3 “Extremely difficult”. No neutral option was used. We then dichotomize the variable (Yes: *Extremely difficult* and *difficult* and No: *Not at all difficult* and *not very difficult*). The variables used to predict the “job difficulty” are the following:

**Information level**: How do you evaluate the level of information given to you by the hospital management on the current epidemic? (Insufficient/Adequate). We deduce from this question the variable Sufficient Information (Yes/No).

**Department change**: Has your hospital changed your place of work during the pandemic? (Yes/no).

**Applause**: Did society’s applauses impact on your motivation to work? (Yes/No).

**Motivation**: What is the most important factor that supports your work motivation while performing this dangerous task? (My Religious Belief/Doctors’ Oath/Family Support/Remuneration/Colleagues Solidarity).

### 2.3. Statistical Data Analysis

First, descriptive statistics were conducted to check the distribution of the work system factors among the different demographic subgroups. Second, a chi-square test was run to calculate the significance of the correlation between the dependent variable of our study (outcome: job difficulty perception) and the demographical characteristics of the population and their psychological wellbeing characteristics. Then, by adjusting the model to the significantly correlated variables, we conducted multivariable logistic regression analysis to check the impact of the independent variables of our models on the dependent variable (Job difficulty perception). A *p* value of (<0.1) was considered significant as it signifies a positive correlation. All data cleaning and analyses were performed using Python 3.7 (The Python Software Foundation (PSF), Hoboken, NJ, United States of America).

In a previous study, we found that job position was correlated with the clinicians’ intention to leave their job [24]. Healthcare providers’ age, gender, job category, and site of practice were found to be important predictors of their experience during the pandemic [26]. Based on the Chi-square test run, we found that only psychological wellbeing variables of the respondents (depression, burnout, and anxiety) correlated with their perception of job difficulty. We, thus, adjust our logistic multivariable model to the covariates that are correlated with the job difficulty perception. This method (adjusted logistic multivariate model) was used in different studies before [24].

## 3. Results

The survey was distributed to (*n* = 700) healthcare workers and (*n* = 345) responses were received. Most of the respondents were female (85.22%). Among these respondents, 20.58% were registered doctors, 28.99% were registered nurses or midwives, and the rest were other health staff as mentioned in Table 1. A majority of 44.93% worked in emergency rooms (ER) and 60.29% of them were younger than 35 years old. A majority of the respondents reported a low depression rate (51.88%), high anxiety (STAI-I: 75.65% and STAI-II: 54.49%), and a high burnout level (51.01%).

Most of the respondents (82.61%) thought the level of information given to them by the hospital management on the current epidemic was enough, and 40.29% of them changed departments during the pandemic. In total, 37.39% of the respondents think that their most important motivation is a religious belief, and 21.74% think they appreciate peer support.

As shown in Table 2, 31.30% of the respondents experienced job difficulty. We explored the correlation between the dependent variable (job difficulty) and the possible predictors and covariates. First, we run the Chi-square test to find the potential covariates to add to our main model.

We also adjusted our logistic multivariable model to the covariates that are correlated with job difficulty perception. The correlated covariates are anxiety, burnout, and depression. All results are summarized in Table 3. Burnout, depression, applause, and department change were significant predictors of the employees’ perception of job difficulty.

Adjusted for demographics and psychological factors, our model improved pseudo R^2^ from 0.03 to 0.20. The model shows that the severe depression and burnout that healthcare professionals were facing during the pandemic were significantly correlated with their perception of job difficulty (OR Severe PHQ-9 = 10.8, *p* = 0.004; OR Severe Burnout = 7.83, *p* < 0.001). The professionals who were changed from one department to another were also more likely to perceive the job as difficult (OR Department Change = 1.60, *p* = 0.045). However, those who thought society’s applause would impact their motivation to work were more likely to think that they did not face any difficulties doing their job during the pandemic (OR Applause = 0.56, *p* < 0.016).

## 4. Discussion

This study was conducted during the first several months of the COVID-19 pandemic in one of the most impacted healthcare systems in a developing country, Turkey. Our study showed that the perception of job difficulty is impacted by both emotional pressures, such as factors that cause burnout and depression, as well as environmental factors, including department change and social support. However, the quantity of information shared, the religious beliefs, the monetary compensation, and the peer support were not associated with the perception of job difficulty among the study group. The COVID-19 pandemic has put front-line healthcare workers in stressful and risky work situations. Difficulties related to the practices can, then, cause medication errors [27] and disturb their emotional well-being [28]. For these reasons, it is important to make sure that healthcare workers have a positive environment and support to maintain their critical work in a pandemic situation.

Work difficulties encountered by health professionals have been categorized into the following three themes: people management difficulties, work and resources constraints, and self-management with decision making under pressure [28]. The outbreak of the COVID-19 disease is not only a health crisis, but it is also a social crisis that affects the public and organizations in various ways. Managing its emotional and psychological effects is vitally important for both individuals and communities [29]. As a result of practices such as quarantines, curfews, social distancing, and staying away from one’s family, healthcare workers have been subjected to psychological stress that has impacted their task performance and social interactions [30]. Among workers in healthcare, moral distress has been associated with concurrent feelings of emotional fatigue and low job satisfaction [31,32].

We found, in our study, that emotional stress (burnout and depression) were significant predictors of job difficulty perception among healthcare workers. Health professionals experienced very severe depression and burnout due to the fear of infection and trauma from the suffering and death events they witnessed [33]. Physio-psychological health and work performance may be adversely affected by persistent exposure to anxiety [34]. In another similar study, it was found that emotional stress impacted healthcare workers’ intentions to leave their jobs [24].

In addition, COVID-19 required new wards to be created in hospitals and changes in the work processes, so many HCWs had to change their departments to better serve the infected patients. There are even reports of HCWs having been relocated to other cities or provinces in many countries, such as China [35]. It is known that changes in the work environment can lead to increased stress among healthcare professionals, especially in such circumstances [36]. In the context of the pandemic, professionals who changed departments were more likely to display decreased mental health. We could prove these mental health issues through the severe burnout and depression rates we found. This can explain how, adjusted for the emotional stress faced, professionals declared that changing departments during COVID engendered negative perceptions towards the ease of doing their jobs. Furthermore, the results showed that monetary compensation and virus-related information availability were not influencing their perception of job difficulty. Social support, however, was reported as one of the primary factors influencing their perception of job difficulty. In fact, it has been proven that people who receive social support perceive themselves as more in control of their lives and can handle uncertainty better [37]. In the case of health professionals, social support was found to be strongly connected to better health outcomes and lower job strain [38,39].

During the pandemic, front-line health care professionals have had challenging and unprecedented experiences, which may negatively impact their performance. Thus, in order to maintain good work performance among healthcare workers, more attention should be paid to the importance of their emotional support. First, organizational support is needed. Hospital managers should work on maintaining their workers’ professional enthusiasm by offering them good work environments. They should be provided with the resources and help needed to fulfill their jobs. Patients can also contribute to the providers’ well-being building by providing positive feedback to keep connections with their doctors and nurses. Finally, even though external help is much appreciated in such circumstances, healthcare professionals’ self-adjustments remain of great importance too.

Doctors, nurses, and health staff should work on reestablishing their motivation to work. Blocking the unpleasant pandemic-related memories and the negative emotions should be the first step to take to be able to make good decisions and overcome the cognitive difficulties and the work stress. To sum up, in order to solve the job difficulty-related issues (people management difficulties, work and resource constraints, and self-management with decision making under pressure), healthcare workers should focus on the following three management dimensions: seeking help from society, organizational support, and self-adjustment measures.

**Figure 1:**
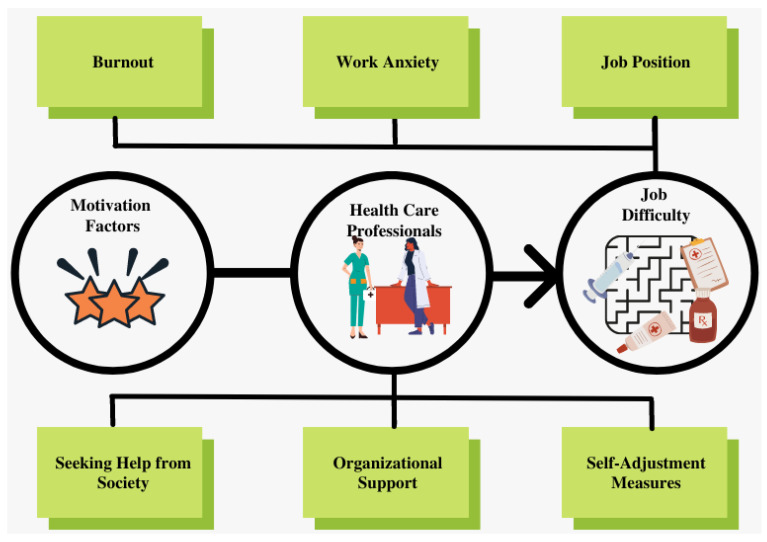
Framework of motivational factors of work environment and solutions to job difficulty perception.

Many studies are investigating the changes from normal life to the pandemic situation to help prevent failures of healthcare systems in future crises [40,41]. However, it remains of some importance to consider the transition from a pandemic situation to normal life. This can help us prevent disruption of system performance and disequilibrium in demand and offer within health care organizations that we want to focus on in future studies to help maintain a smooth post-COVID transition. Figure 1 shows a framework explaining job difficulty and proposed solutions.

There are a few limitations of the study. First, this is a cross-sectional study with the participants from one hospital in Turkey, which might limit the generalization, despite the fact that we know that the findings and insights are supportive of healthcare systems worldwide. Second, the voluntary nature of the survey may have resulted in selection bias and response bias. Respondents experiencing work-related issues may have been more likely to participate in the survey, leading to overreporting. Future studies should also expand the findings of these results to generalize the findings in different settings and circumstances. Third, the study was designed for a specific culture. The motivations included were (religion, financial, and social). Although the findings are limited to the hospital that the study was part of, the insights are supportive of healthcare systems worldwide. Replicating this study in other settings, for generalizability purposes, should consider the possible motivators of the targeted population (e.g., work ethics, political thoughts, etc.). 

## 5. Conclusions

With the work system disruptions, the COVID-19 pandemic is deranging the emotional well-being of healthcare professionals. Our study showed that healthcare professionals were experiencing high burnout and depression rates during the first wave of the COVID-19 pandemic. Such pressures impacted the healthcare professionals’ perception of job difficulty. This study showed that factors such as (applause and department change), job load, and mental well-being (depression and burnout) influence job difficulty perception. Due to the significant shortage of frontline professionals in the global healthcare industry, efforts need to be made to provide more technical and motivational support to healthcare professionals. Future studies should specifically explore each factor influencing job description with a longitudinal study to see the effects on the perception of job difficulty as well as the intention to leave the job in the long term in various settings and circumstances.

## Figures and Tables

**Table 1:** Descriptive statistics of the work factors’ distribution of the respondents.

Variables		% (*n*)
Sex	Male	14.78% (*n* = 51)
	Female	85.22% (*n* = 294)
Education	Middle school	66.38% (*n* = 229)
	High school	16.23% (*n* = 56)
	University	7.25% (*n* = 25)
	Doctorate	10.14% (*n* = 35)
Job Position	Health staff	20.00% (*n* = 69)
	Nurse	28.99% (*n* = 100)
	Doctor	20.58% (*n* = 71)
	Other	30.43% (*n* = 105)
Department	ER	44.93% (*n* = 155)
	Inpatient Unit	24.64% (*n* = 85)
	ICU	6.38% (*n* = 22)
	Other	24.06% (*n* = 83)
Age	HCWs less than <34 years old	60.29% (*n* = 208)
	HCWs more than ≥35 years old	39.71% (*n* = 137)
PHQ-9	Low	51.88% (*n* = 179)
	Moderate	28.70% (*n* = 99)
	High	19.42% (*n* = 67)
Anxiety1	Low	8.70% (*n* = 30)
	Moderate	15.65% (*n* = 54)
	High	75.65% (*n* = 261)
Anxiety2	Low	18.55% (*n* = 64)
	Moderate	26.96% (*n* = 93)
	High	54.49% (*n* = 188)
Burnout	Low	15.07% (*n* = 52)
	Moderate	33.91% (*n* = 117)
	High	51.01% (*n* = 176)
Sufficient Information	No	17.39% (*n* = 285)
	Yes	82.61% (*n* = 60)
Department Change	No	59.71% (*n* = 206)
	Yes	40.29% (*n* = 139)
Applause	No	41.45% (*n* = 143)
	Yes	58.55% (*n* = 202)
Motivation	Oath	10.72% (*n* = 37)
	Religious beliefs	37.39% (*n* = 129)
	Peer support	21.74% (*n* = 75)
	Family support	17.97% (*n* = 62)
	Monetary compensation	12.17% (*n* = 42)

**Table 2:** Correlation between the demographic and psychological variables and perception of job difficulty.

Variables		Job Is Difficult		Total All	*p*-Value
		No	Yes		
Sex	Male	64.71%	35.29%	14.78%	0.507
	Female	69.39%	30.61%	85.22%	
Education	Middle school	65.94%	34.06%	66.38%	0.293
	High school	76.79%	23.21%	16.23%	
	University	72.00%	28.00%	7.25%	
	Doctorate	71.43%	28.57%	10.14%	
Job Position	Health staff	67.82%	32.18%	50.43%	0.151
	Nurse	62.00%	38.00%	28.99%	
	Doctor	80.28%	19.72%	20.58%	
	Other	68.67%	31.33%	24.06%	
Department	ER	68.39%	31.61%	44.93%	0.744
	Inpatient Unit	67.06%	32.94%	24.64%	
	ICU	77.27%	22.73%	6.38%	
Age	Younger population (≤34)	66.83%	33.17%	60.29%	0.358
	Older population (≥35)	71.53%	28.47%	39.71%	
PHQ-9	Low	79.89%	20.11%	51.88%	0.00 ***
	Moderate	76.77%	23.23%	28.70%	
	High	26.87%	73.13%	19.42%	
Anxiety1	Low	83.33%	16.67%	8.70%	0.01 *
	Moderate	75.93%	24.07%	15.65%	
	High	65.52%	34.48%	75.65%	
Anxiety2	Low	85.94%	14.06%	18.55%	0.00 ***
	Moderate	80.65%	19.35%	26.96%	
	High	56.91%	43.09%	54.49%	
Burnout	Low	90.38%	9.62%	15.07%	0.00 ***
	Moderate	80.34%	19.66%	33.91%	
	High	54.55%	45.45%	51.01%	
Total		68.70%	31.30%	NA	

*p < 0.05, *** *p* < 0.001.

**Table 3:** Results of the multivariable logistic regression of the perception of job difficulty.

		Odds Ratios (OR)	*p*-Value
PHQ-9	Low	NA	
	Moderate	10.8 (5.73–21.22)	0.00 **
	Severe	1.04 (0.40–2.69)	0.93
Burnout	Low	NA	
	Moderate	2.30 (0.88–7.18)	0.112
	Severe	7.83 (3.24–23.39)	0.00 ***
Applause	No	NA	
	Yes	0.56 (0.35–0.90)	0.016 *
Department change	No	NA	
	Yes	1.60 (1.00–2.54)	0.045 *

* *p* < 0.05, ** *p* < 0.01, *** *p* < 0.001, OR: Odds Ratios, The format of the odds ratios is OR (95% CI), CI: Confidence Interval.

## Data Availability

The data presented in this study are available on request from the corresponding author.
